# Novel Investigational Agents and Pathways That May Influence the Future Management of Acute Myeloid Leukemia

**DOI:** 10.3390/cancers15112958

**Published:** 2023-05-29

**Authors:** Naveen Premnath, Yazan F. Madanat

**Affiliations:** 1 Division of Hematology and Medical Oncology, Department of Internal Medicine, University of Texas Southwestern Medical Center, Dallas, TX 75235, USA; naveen.premnath@utsouthwestern.edu; 2 Harold C. Simmons Comprehensive Cancer Center, UT Southwestern Medical Center, Dallas, TX 75235, USA

**Keywords:** AML, leukemia, NK cell, menin, immunotherapy, cell therapy

## Abstract

**Simple Summary:**

Leukemia or blood cancer has been treated with chemotherapy since the 1970s. The treatment had undergone little change until the last decade when many new molecules were approved. Many different targeted agents that are effective in subgroups of leukemia targeting specialized pathways or in patients with specific mutations are now available. Classification systems have advanced with time from being based on the appearance of leukemia underneath the microscope to now being based on the presence of mutations and other genetic characteristics. We have explored multiple different approaches to fighting leukemia based on modulating and priming the immune system or with the help of cellular therapy and bone marrow transplants. Many newer pathways to targeting cancer are being tested in the laboratory and are being brought to the bedside through clinical trials. This is an exciting decade for researchers and clinicians and many major changes are expected to unravel in the treatment of leukemia.

**Abstract:**

Acute Myeloid leukemia (AML) is a clinically heterogeneous disease with a 5-year overall survival of 32% between 2012 to 2018. The above number severely dwindles with age and adverse risk of disease, presenting opportunities for new drug development and is an area of dire unmet need. Basic science and clinical investigators across the world have been working on many new and old molecule formulations and combination strategies to improve outcomes in this disease. In this review, we discuss select promising novel agents in various stages of clinical development for patients with AML.

## 1. Introduction

Acute myeloid leukemia (AML) is an aggressive hematological malignancy that has been at the forefront of investigational agent development. Immature myeloid precursor cells proliferate rapidly, causing varied symptoms due to decreased production of normal hematological cells and increased immature cells. Immature myeloid cells are sticky and increased numbers can cause decreased tissue perfusion. Since the 1970s, conventional chemotherapy followed by post-remission therapy with chemotherapy combinations of cytarabine has been the backbone of treatment for acute myeloid leukemia. A deeper understanding of the molecular landscape of AML from the knowledge gained from routine next-generation sequencing employed in clinical practice and research settings has paved the way for the identification of key genetic changes and molecular mutations that drive leukemia. By selectively targeting aberrant molecular events, these investigational agents offer the potential for enhanced efficacy and reduced toxicity compared to conventional chemotherapy. We have seen a total of 10 new agents approved for acute myeloid leukemia over the last decade and many more agents that are promising in the pipeline. These agents include midostaurin, enasidenib, ivosidenib, olutasidenib, venetoclax, gemtuzumab, glasdegib, gilteritinib, oral 5-azacitidine, and CPX-351.

Despite advances in therapy, the overall prognosis for AML remains poor, with high relapse rates and limited treatment options among patients with complex karyotypes and poor risk mutations who are resistant to conventional chemotherapy. Non-intensive induction strategies with comparable remission rates to conventional chemotherapy such as the combination of a BCl-2 inhibitor and a hypomethylating agent [1] have posed newer questions to clinicians if older individuals should be the only groups to receive non-intensive induction or if these strategies need to explored in groups known to be resistant to conventional chemotherapy. However, any upfront randomized controlled trials comparing conventional vs. less intensive therapy are lacking and decisions are made based on expert opinions. Strategies to identify biomarkers or other features among the leukemia cells which can predict responses to conventional chemotherapy vs. less intensive therapy are being explored [2]. Limitations of conventional chemotherapy like organ toxicities, risk of early induction mortalities, and worsening immune dysfunction are in fact highlighted as advantages with targeted agents.

**Figure 1:**
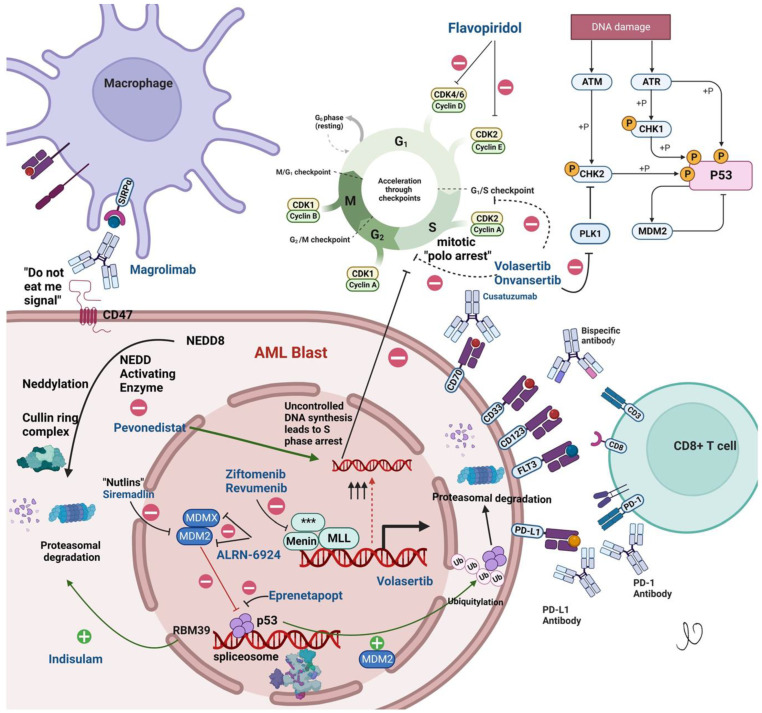
Novel investigational agents. ATM—ataxia telangiectasia mutated kinase, ATR—ataxia telangiectasia and Rad3 related, CDK—Cyclin dependent kinase, CHK—check point kinase, FLT3—FMS like tyrosine kinase, MDM—Mouse Double Minute, NEDD8—Neural cell developmentally downregulated 8, PD—L Programmed cell death ligand, PLK—Polo like Kinase, RBM-RNA-binding motif protein, SIRPα- signal regulatory protein alpha, ***—variable fusion partner.

Some strategies that have been gaining traction include empowering the body’s own immune system using immune checkpoint inhibitors, cellular therapies such as chimeric antigen receptor T-cell (CAR-T cells), and modulation of the innate immune system through Natural Killer (NK) cells. Differentiation agents that force immature AML cells to mature and small molecule targets identified through reverse engineering which provide a survival advantage to leukemia clones are all also being evaluated. Although decreased risks of cytokine release syndrome and neurotoxicity among frailer patients have been quoted as advantages of NK cell therapy, its inability to overcome the immune-suppressive tumor microenvironment has prevented this strategy from producing durable remissions and efficacy [3]. Similarly, the development of newer mutations that provide escape mechanisms and decreased dependence on targeted pathways have plagued small molecule inhibitors [4]. Figure 1 shows selected promising agents currently in different stages of development and highlights their mechanisms of action. Select investigational agents currently in phase III of clinical trial development for patients with AML are summarized in Table 1.

## 2. Menin Inhibitors

Originally described in the context of multiple endocrine neoplasia 1 (MEN1) as a tumor suppressor, it was later found that menin regulates hematopoiesis and myeloid transformation. Chen et al. described the role of menin in normal bone marrow hematopoiesis and the proliferation of MLL-AF9 mutated leukemic cells [5]. Menin interacts with lysine methyl transferase 2A *KMT2A*, previously known as mixed lineage leukemia 1 or the MLL1 gene and has a paradoxical oncogenic role in the proliferation of *KMT2A*-rearranged leukemias. MLL1 or *KMT2A* translocations, found in about 5–10% of all adult AML cases, lead to a fusion protein between *KMT2A* and about 80 different fusion partners, all of which bind to menin at the N- terminus end of *KMT2A*. This interaction between menin and the *KMT2A* fusion protein is essential for leukemic transformation by increasing homeobox A9 (HOXA9) gene expression [6]. Nucleophosmin (*NPM1*) gene mutations are common and occur in 30% of all reported AML cases. *NPM1* mutations lead to increased HOX pathway expression and require the interaction between *KMT2A* and menin for proliferation. Disruption of this interaction leads to differentiation in *NPM1*^mut^ AML [7]. These findings became the preclinical basis for trials that led to the use of small molecule menin inhibitors in *KMT2A*-rearranged and *NPM1*^mut^ AML cell lines [8] and thereafter in clinical trials. The American Society of Hematology (ASH) meeting in December 2022 featured data from two phase 1 clinical trials using menin inhibitors in *NPM1* mutated/*KMT2A*-rearranged AML that showspromising results. Issa et al. reported updated results from the AUGMENT-101 phase 1 trial in 68 patients with heavily pretreated (median 4 lines) R/R AML who received the menin inhibitor SNDX-5613 (now known as revumenib) with dose escalation [9]. The medication was well tolerated with 16% of patients developing differentiation syndrome (DS), none of which were grade 3 or higher. Grade 3 or higher adverse events were seen in less than 5% of patients. Importantly, during dose escalation, some patients experienced asymptomatic grade III QTc prolongation. The overall response rate (ORR) was 53% with 20% of patients achieving complete remission (CR). Another menin inhibitor KO-539 (known as ziftomenib) is also being tested in the phase 1/2 KOMET-001 trial as an oral once-daily pill [10]. ORR of 42% was reported with good tolerability and a CR rate of 16.7%. Interestingly, patients who experienced DS had an ORR of 75%. Many groups have already provided preclinical evidence for combinations of menin inhibitors with agents like BCL-2 inhibitors, CDK6 inhibitors and DOT1L inhibitors, and FLT3 inhibitors [11,12,13]. Given the promising results, a few other menin inhibitors are currently in development as single agents and using combination strategies.

## 3. Anti-CD47 Antibody

CD47 is a transmembrane protein expressed on a wide array of cells and interacts with its ligand signal regulatory protein alpha (SIRPα) on macrophages to inhibit phagocytosis, now widely known as a “don’t eat me” signal [14]. Work from the Weissman lab originally described the upregulation of these molecules on hematopoietic stem cells (HSC) and leukemia stem cells (LSC) [15] to escape immune surveillance. They also described that in most AML cases, the LSCs have higher expression compared to HSCs and can be targeted with monoclonal antibodies to induce phagocytosis [16]. Majeti’s group was able to successfully make a humanized anti-CD47 antibody and was able to demonstrate its efficacy in vitro and in mouse models [17]. This Hu5F9-G4 antibody, now better known as magrolimab, is only one of the many anti-CD47 antibodies in clinical trials. During the initial development of CD47 monoclonal antibodies, many studies were suspended due to toxicities/adverse events. The first phase 1 study CAMELLIA, launched in the UK, was terminated due to severe anemia [18]. It is now known that CD47 is commonly expressed on red blood cells (RBC). It is a senescence marker of RBCs as it is lost in aged cells leading to their increased destruction. Most patients were found to develop anemia during this trial and the blood bank ran into issues with RBC compatibility testing [19]. Later, with a priming strategy called “pruning” by using a low dose of the drug a week before the actual dose, they were able to remove the older RBCs. This generated many reticulocytes that were rich in CD47 and led to better tolerability of therapeutic doses with less anemia [20]. Zeidan et al. published their data from the phase 1 trial that evaluated an anti-CD47 monoclonal antibody CC-90002 in 28 patients with AML (24) and high-risk MDS (4) and reported common side effects of diarrhea, hematological toxicities, and liver function test abnormalities [21]. However, patients who received CC-90002 did not report any objective responses. Another strategy that led to further improvements in anti-47 therapy was through combination with other molecules, which helped unmask or increase pro-phagocytic signals in leukemia cells in the setting of removal of the “do not eat me” signal. Calreticulin is one such pro-phagocytic signal and its expression is upregulated by exposure to azacitidine in MDS and MPN cell lines [22]. Clinical benefit has been demonstrated in patients with *TP53*-mutated AML. In this phase 1b study, using 75 mg/m^2^ azacitidine on days 1 to 7 of a 28-day cycle combined with a magrolimab priming dose of 1 mg/kg followed by a ramp up to 30 mg/kg weekly or fortnightly for patients with newly diagnosed *TP53*-mutated AML, the ORR was 48.6% in 72 patients with p53-mutated AML [23]. The median overall survival reported with limited follow-up was 10.8 months. Two ongoing phase III clinical trials investigating magrolimab in AML are underway. ENHANCE-2 is a randomized open-label study, evaluating the combination of magrolimab with azacitidine vs. physician’s choice (intensive vs. less intensive approach) in previously untreated patients with *TP53*-mutant AML. ENHANCE-3 is a phase 3, randomized, double-blind, placebo-controlled study of magrolimab vs. placebo in combination with venetoclax and azacitidine in newly diagnosed AML. Many other CD-47-related molecules are in different stages of clinical development either in combination with chemotherapy, monoclonal antibodies, or immune checkpoint inhibitors.

## 4. Polo-Like Kinase Inhibitors

Numerous signaling pathways work together to form checkpoints during cell division, especially during the transitions between the G1 and S phases and G2 and M phases. Serine-threonine kinases like cyclin-dependent kinase (CDK), Polo-like kinase (PLK), and Aurora kinases are involved in regulating mitosis at different levels [24]. Five different PLKs have been described, of which PLK1 is the best studied and well-known. PLK plays a critical role in mitosis by controlling centrosome maturation, spindle formation, and cytokinesis, and its dysregulation leads to either a mitotic “polo arrest” or accumulation of genetic instability, aneuploidy, a hallmark of cancer [25]. PLK1 also controls entry into the mitotic cycle post DNA damage and, by causing inactivation or degradation of p53, can work as a mitotic proto-oncogene [26]. Renner et al. demonstrated that AML cell lines overexpress PLK1 compared to CD34+ stem cells and inhibition with PLK1 led to decreased proliferation of blasts without affecting HSCs [27]. PLK contains two C-terminal polo-box domains and an N-terminal ATP binding site targeted by PLK1 inhibitors like volasertib, which competitively bind to the ATP binding site and inhibit mitosis [28]. However, the large phase 3, double-blind, placebo-controlled trial that used a combination of volasertib IV 350 mg on days 1 and 15 along with low dose cytarabine 20 mg IV on days 1 to 10 of a 28-day cycle in 666 patients over 65 years old with newly diagnosed AML who were not eligible for intensive induction did not meet its primary endpoint ORR [28]. The study arm had an ORR of 25.2% compared to the placebo and low-dose cytarabine arm 16.8% (*p* = 0.071). OS was in fact lower with volasertib 5.6 months vs. 6.5 months (*p* = 0.757), likely owing to the increased grade ≥ 4 cytopenia and infections and increased death observed with volasertib. Factors like an extremely long half-life of 5 days and lack of PLK1 selectivity are being offered as possible explanations for its failure. Zeidan et al. reported that the oral, more selective PLK1 inhibitor onvansertib with either low-dose cytarabine 20 mg/m^2^ on days 1 to 10 or decitabine 20 mg/m^2^ on days 1 to 5 in R/R AML was well tolerated in their phase 1b study [29]. The phase 2 part of this study has completed recruitment and results are being analyzed. Overexpression of PLK1 is clearly a poor prognostic factor [30] and is more prevalent in subtypes of AML [31] such as ones with complex karyotypes [32]. We believe better patient selection and development of novel biomarkers for response prediction or novel delivery systems like transferrin-guided intelligent nanovesicles [33] could possibly influence the future of PLK1 inhibitors.

## 5. NAE Inhibitors

Proteasome inhibitors that target the ubiquitin-proteasome system are used in many malignancies. In multiple myeloma, they prevent the degradation of intracellular proteins. This leads to the accumulation of these proteins that trigger a cascade of events that lead to cell death. Neural cell developmentally downregulated 8 (NEDD8) is a protein homologous to ubiquitin and NEDD-activating enzyme (NAE) leads to neddylation and thereby degradation of certain proteins [34]. However, in contrast to proteasome inhibition, NEDD8 pathway inhibition does not affect intracellular protein turnover but rather leads to uncontrolled DNA synthesis, DNA damage, and arrest in the S phase followed by cell death [34]. Based on early signals regarding dependence on the NEDD pathway in AML, Swords et al. successfully demonstrated the activity of a novel inhibitor of NAE in mouse models and proposed a mechanism that induces apoptosis [35]. Later, their group reported phase 1b tolerability data using the same inhibitor, now called pevonedistat, IV 20 to 30 mg/m^2^ on Days 1, 3, and 5 with azacitidine 75 mg/m^2^ on Days 1 to 5, 8, and 9 every 28 days. Grade 3 anemia and neutropenia were noted in 30% of patients along with increased liver function tests in 6% of patients with newly diagnosed AML unfit for intensive induction aged more than 60 years [36]. An encouraging ORR of 50% was seen in this cohort. Unfortunately, a phase 3 combination of pevonedistat and azacitidine in MDS, CMML, and low blast count AML did not meet its primary endpoint [37]. Based on preclinical data that suggested that the combination of pevonedistat and azacitidine increased sensitivity to venetoclax [38], two separate groups have now presented their phase 1 data on this triplet combination and have started phase 2 recruitment. Guru Murthy et al. in their phase 1 PAVE (Pevonedistat, Azacitidine, and Venetoclax) trial recruited 16 patients with R/R AML and established 20 mg/m^2^ as the phase 2 dose for pevonedistat and reported a to-date ORR of 40% [39]. Short et al. used the triplet in 28 newly diagnosed AML patients unable to get intensive induction therapy with an ORR of 71% and a more impressive ORR of 63% in patients with poor risk cytogenetics [40]. However, the combination was quite myelosuppressive and led to infection/neutropenic fevers in 61%. Reassuringly, the 4-week and 8-week mortalities were only 7% and 14%, respectively, in this debilitated population. Another unique side effect was hypophosphatemia, noted in 29% of the patients. Given the negative phase III clinical trial results, there are no trials currently accruing using pevonedistat and many trials have been terminated.

## 6. The Mouse Double Minute 2 (MDM2) Pathway

TP53 mutations are uncommon in AML accounting for 6–8% of all cases. However, overexpression of MDM2, a negative regulator of the p53 axis [41] causing wild-type p53 inactivation, is seen in as many as 50% of all AML cases [42]. Vassilev et al. first described a class of small molecule inhibitors and named them “Nutlins” after their town Nutley in New Jersey. Nutlins inhibited p53-MDM2 binding, leading to upregulation of p53 through a posttranslational mechanism and demonstrating its potency in inhibiting tumor xenografts in nude mice [43]. The first MDM2 inhibitor RG7112 showed activity in a phase 1 study but also revealed major toxicities, mostly gastrointestinal, with grade 4 toxicities in 30% of patients leading to the abandonment of this molecule [44]. Unfortunately, the phase 3 placebo-controlled MIRROS trial that evaluated cytarabine 1 g/m^2^ IV on days 1 to 5 with or without idasanutlin 300 mg, PO BID daily in 28-day cycles in R/R AML did not meet its primary endpoint OS [45]. The medication caused diarrhea in 80% of the patients and nausea in 44%, with slightly higher but comparable hematologic toxicities as single agent cytarabine. A pegylated prodrug of idasanutlin has not shown an improved safety profile [46]. Siremadlin, another MDM2 inhibitor, has however demonstrated a better safety profile with lower rates of diarrhea (13%) but did report cytopenia and nausea [47]. A promising molecule ALRN-6924, which is the first ever “stapled peptide” stabilized in an alpha-helical configuration able to bind to both Murine double minute-X MDMX and MDM2, two potent inhibitors of p53, was tested in phase 1 back in 2018, however no information is currently available regarding its progress in AML [48]. The dual inhibitor had much better GI tolerance and lesser toxicities in general. However, the parent company has likely switched gears and the molecule is now being actively tested as an agent that prevents chemotherapy-induced myelosuppression. A few more MDM2 inhibitors alone or in combination are being evaluated in early phase trials in hopes of better tolerance. Due to the upregulation of MDM2 with reversible small molecular inhibitors, novel MDM2 degraders are being developed. KT-253 is one such highly potent heterobifunctional MDM2 degrader that has demonstrated promising activity in preclinical models [49].

## 7. P53 Reactivators

TP53 is a tumor suppressor gene and mutations in TP53 in AML often cause poor outcomes. The international consensus classification (ICC) now has *TP53*-mutated AML as a separate entity under “myeloid neoplasms with mutation *TP53”* to delineate the poor outcomes and highlight the need for better therapies irrespective of blast percent [50]. The French group GFM reported the phase 2 results [51] of eprenetapopt APR-246, a novel first-in-class small molecule reactivator of mutant p53. Eprenetapopt is the methylated form of PRIMA-1 (PRIMA-1^MET^) which has been shown to covalently bind to a mutated p53 protein and restore its wild-type conformation. It also increases its proapoptotic activity via induction of Bax and thereby caspase activation [52]. Maslah et al. had previously demonstrated the synergistic effects of combining APR-246 with azacitidine (AZA) in p53-mutated MDS and AML cell lines as well as in a xenotransplant model [53]. In this phase 2 study, 52 patients with p53-mutated AML (n = 18) and MDS (n = 34) were treated with eprenetapopt 4500 mg IV on Days 1 to 4 combined with azacitidine 75 mg/m^2^ subcutaneous injections daily from days 4 to 10 of a 28-day cycle followed by consolidation with allogenic SCT. The same combination was offered at a lower dose as a maintenance strategy. Common side effects noted were febrile neutropenia (37%) and neurological adverse events (40%). Grade 3 neurological events were reported in only 6% and were all reversible with drug discontinuation and showed no recurrence when the drug was resumed with dose reduction. Efficacy analysis showed an ORR of 33% in AML patients and 62% in MDS patients. Another parallel study from the US reported their combined results with the French GFM group at the ASH meeting in 2021 with no additional safety signals noted [54]. Mishra et al. evaluated the use of eprenetapopt IV 3.7 g once daily on days 1 to 4 with azacitidine injections 36 mg/m^2^ once daily from days 1 to 5 of a 28 day cycle in *TP53*-mutated AML and MDS patients as a post-transplant maintenance regimen and reported that the 1-year RFS of 60% has met their prespecified hypothesis of ≥50%, as historical data correspond to 30% RFS [55]. A phase 3 trial using eprenetapopt in patients with *TP53* mutant MDS failed to meet its primary endpoint CR [56]. In the intention-to-treat population of 154 patients, the CR rate in the eprenetapopt with AZA arm was 33.3% (95% CI: 23.1–44.9%), compared to 22.4% (95% CI: 13.6–33.4%) in the AZA-alone arm (*p* = 0.13). As a result, the company halted further development of this agent.

## 8. Cell Cycle Inhibitors

### Cyclin-Dependent Kinase (CDK) Inhibitors

Cyclin-dependent kinases are serine-threonine kinases that act as important checkpoints in the cell cycle. Flavopiridol, also called alvocidib, a multi-CDK inhibitor, has activity against CDK4, 7, and 9 and leads to apoptosis of leukemia cells primarily due to its effects on transcriptional regulation due to CDK9 inhibition [57]. Alvocidib’s anti-tumor effects are accentuated when administered prior to cell-specific cytotoxic agents. FLAM is a combination of agents (flavopiridol, cytarabine, and mitoxantrone) that utilizes this timed sequential therapy (TST). Following good tolerability in phase 1 studies, Zeidner et al. conducted a randomized phase 2 trial of FLAM. Flavopiridol 50 mg/m^2^ IV administered on days 1 to 3 followed by cytarabine 2 g/m^2^ IV on days 6–8 and mitoxantrone IV 40 mg/m^2^ IV on day 9 versus 7 +3 in 165 patients with newly diagnosed AML led to an increased CR rate of 70% with the TST with FLAM, compared to 46% with 7 + 3 induction (*p* = 0.003) [58]. However, the study was not powered enough and did not translate into an OS difference. The tolerability of FLAM was comparable to standard 7 + 3 as there was no difference noted in grade ≥ 3 toxicities between the two groups. The study also showed a better response in secondary AML patients even though the cohort was small. A phase 1 study of alvocidib followed by 7 + 3 has also been reported with good tolerance when tumor lysis is anticipated and acted on in a timely manner [59].

## 9. Immunotherapies

### 9.1. Antibodies

Advancement in multiparametric flow cytometry and immunohistochemistry has led to the recognition of multiple surface antigens that help distinguish blast cells and leukemia stem cells from other progenitors and mature blood cells. Monoclonal antibodies directed against these antigens are able to activate immune effectors via the complement system, leading to antibody-dependent cellular cytotoxicity which leads to phagocytosis by antigen-presenting cells, resulting in tumor antigens being presented on major histocompatibility complex (MHC) that activate CD8+ve cytotoxic T cells.

### 9.2. CD33-Antibody Drug Conjugate

Vadastuximab was a CD33-directed antibody conjugated to pyrrolobenzodiazepine that, post successful early phase trials, underwent a phase 3, double-blind, placebo-controlled CASCADE trial where vadastuximab was given with a hypomethylating agent (azacitidine or decitabine) in comparison to only HMA in older patients with newly diagnosed AML [60]. However, the trial was discontinued based on the independent drug monitoring committee’s finding of an increased rate of deaths secondary to infections in the study arm.

### 9.3. CD33xCD3 Bispecific T Cell Engager (BiTE) Antibody AMG330

Bispecific engagers help bring effector cells (cytotoxic T cells, NK cells, or macrophages) using the surface antibody part of their construct directly to the tumor cells recognized by a second antibody. In a phase 1 trial, AMG330 was given as a continuous infusion in 55 patients with R/R AML in a dose-step-up approach with dexamethasone prophylaxis [61]. Cytokine release syndrome was noted in 67% of the patients, with grade 3 or higher in 15% of patients. In this heavily treated population, 17% of evaluable patients achieved CR/CRi. Unlike previous BiTE constructs, AMG 673 (now called Emerfetamab) fused a single chain IgG Fc region to their CD33xCD3 construct to obtain an extended half-life product. This was tested in a phase 1 trial as two infusions on days 1 and 5 of a 14-day cycle [62]. Thirty patients tolerated the treatment with about 50% CRS reported, of which 12% was grade 3 with no grade 4 reported. Another novel construct, the AMV564, a bivalent bispecific CD33xCD3 T cell engager, enrolled 36 patients and did not report any grade 3 or higher CRS, with the most common grade ≥ 3 side effect being anemia in 11% of the patients [63]. An old strategy of using Alpha-emitting particles tagged to an antibody that directs them to targets to avoid collateral damage, given the short range of activity for alpha particles, is making a comeback in the form of Lintuzumab, a CD33 monoclonal antibody tagged to Actinium^225^ (named Actimab-A). This was tested in a phase 1/2 trial in R/R AML given on Day 8 following a salvage regimen of CLAG-M and reported safety data on 21 patients [64]. Dose-limiting toxicities included severe mucositis and delayed neutrophil count recovery (>42 days). The combination is being tested in a larger confirmatory clinical trial.

### 9.4. CD123

A novel Dual Affinity Retargeting (DART) structure was utilized in the making of flotetuzumab, a bispecific antibody with CD3 and CD123 as targets. Uy et al. brought this molecule to clinical practice in their phase 1 trial on R/R AML patients [65]. Safety analysis on 88 patients showed that even though almost every patient (96%) who received the recommended phase 2 dose had an infusion related reaction or CRS, only 8% of them were grade 3 or higher. The only grade 3 or higher side effect occurring in more than 10% of patients was thrombocytopenia (12%). They report that compared to the overall cohort, in patients with primary induction failure or early relapse before 6 months, the ORR was higher at 30%. Studies have suggested that the response to flotetuzumab can be predicted based on Interferon-γ-related gene expression [66]. Vibecotamab is another CD123xCD3 construct currently in phase 2 after good tolerance reported in phase 1 studies [67]. An antibody-drug conjugate construct of CD123 called IMGN632 or pivekimab, using indolinobenzodiazepine as the payload, was used as a triplet combination following early phase single-agent safety studies and updated results were presented at ASH 2022. Pivekimab was given as a single dose on Day 7 along with azacitidine 75 mg/m^2^ on days 1 to 7 and venetoclax 400 mg in 71 patients and had an ORR of 51%. Common side effects reported include febrile neutropenia in 30% of patients, pneumonia in 16%, and grade 3 or higher infusion-related reactions in 3%. APVO436 is a novel bispecific anti-CD123x anti-CD3 ADAPTIR molecule. Results from the expansion phase study in AML and MDS were presented at the ASH 2022 meeting, demonstrating the safety of APVO436 as a single agent and in combination across four different cohorts [68]. This was followed by a press release demonstrating 100% clinical benefit from patients treated in cohort 2 (APVO436 plus venetoclax and azacitidine) for venetoclax-naive patients. Therefore, a planned phase II study of this triplet combination is underway [69].

### 9.5. CD70

Tumor necrosis factor uses CD70 as its ligand. CD70 is expressed on AML LSC and is not present in normal cells or HSCs. Cusatuzumab is a monoclonal antibody against CD70 with enhanced antibody-dependent cellular cytotoxicity. Based on preclinical studies that showed HMA treatment increased expression of CD70 on LSC, Riether et al. conducted a phase 1 trial combining cusatuzumab with azacitidine 75 mg/m^2^ on days 1 to 7 of a 28-day cycle [70]. In 12 newly diagnosed AML patients unfit for intensive chemotherapy involved in the initial report of the phase 1 study, 10 (80%) had achieved CR/CRi and 44% achieved MRD-negativity by flow cytometry. No new signals were reported with long-term exposure to custauzumab for more than 6 months. Novel constructs with FLT3 targets are still in the preclinical stages [71].

## 10. Splicing Modulators

Mutations in the spliceosome complex are commonly found in myeloid malignancies and account for about 30% of all mutations seen in AML [72]. Given the strong mutual exclusivity seen with splicing mutations in AML, it is hypothesized that they are dependent on other splicing factors and a synthetic lethality can be induced by targeting splicing factors. SF3B1 is a splicing factor and an oral modulator of SF3B1 has now entered phase 1 trials. In a mixed cohort of MDS, CMML, and AML patients with 38 AML patients, diarrhea (42%), nausea (28%), and fatigue (17%) were the common side effects noted [73]. Unfortunately, no complete or partial responses were noted in this cohort. Indisulam is an Aryl sulfonamide drug that controls splicing by causing degradation of RNA-binding motif protein 39 (RBM39) [74]. In a phase 2 study in combination with idarubicin and cytarabine that enrolled 39 AML patients, the most common grade 3 or higher side effect was electrolyte abnormalities [75]. Other common grade 3 side effects in this cohort of R/R AML patients were febrile neutropenia (28%), pneumonia (18%), and skin-soft tissue infections (18%). The overall response rate seen in this trial of about 35% is impressive for multiple reasons as the mechanism of action of indisulam was not well known when this trial was performed, nor did they know how to screen for patients who might respond better to indisulam. Future trials incorporating biomarker-based strategies later described by Nijhawan’s group [76] to screen and better identify the cohort of AML patients who would respond to indisulam are awaited. Fong et al. described another potential way to induce synthetic lethality in splicing factor mutant AML through the inhibition of Protein arginine methyl transferase (PRMT) [77]. PRMT inhibitors have already entered phase 1 clinical trials and results are awaited [78]. It is exciting to see preclinical evidence for combining splicing modulators with BCL2 inhibitors [79] and multiple new combination strategies are expected.

## 11. Immunotherapy

### Checkpoint Inhibitors

The widespread success of immunotherapy in multiple solid tumors such as melanoma and renal cell carcinoma led researchers to explore the tumor microenvironment in AML. Based on solid tumor cancer data, they agreed that bone marrow and not peripheral blood would be representative of the tumor microenvironment. Williams et al. first described the immune cell makeup in the bone marrow of AML patients compared to age-controlled healthy donors (HD) [80]. They found that there was no difference in the absolute number of CD3 +ve cells per microscopic field in the bone marrow biopsies between the two groups. However, when CD45^hi^ cells representative of the bone marrow population was sorted by flow cytometry, the CD3 +ve cells were higher in the relapsed AML vs. new AML vs. HD as 81.1% vs. 78% vs. 60.3% (*p* = 0.02), but no differences were found in the CD4 +ve or CD8 +ve T cell subsets. They also noticed a significant difference in the immunosuppressive T regulatory cells in relapsed AML vs. new AML vs. HD as 3% vs. 2.1% vs. 1.7% (*p* = 0.02). Other markers often used as a surrogate for responses to checkpoint inhibitors like PD1-positive CD4 or CD8 T cells were higher in the order relapsed AML > newly diagnosed AML > HD (*p* < 0.01). They also noted that in the subset of *TP53*-mutated patients, there was a higher frequency of PD-L1 +ve blasts compared to AML patients with wild-type *P53*. This finding was further explored in the study conducted by Sallman et al. in 103 MDS or AML patients using their bone marrow mononuclear cells [81]. Using flow cytometry, they showed that P53-mutated AML patients had a higher HSC population and specifically higher PDL1+ve cells among the HSCs (*p* < 0.01). Sallman’s group also measured the MYC expression in relation to the PD-L1 positivity and found a positive correlation (*p* = 0.004, r = 0.615) and also found lower expression of microRNA MiR-34a in the TP53-mutant group (*p* < 0.01). This further supports the hypothesis put forth by Williams et al. that P53 regulates PDL1 expression through miR-34a which first influences MYC expression [80]. The increased expression of immunosuppressive cells seen in p53-mutant AML when combined with these findings suggests that both relapsed AML and p53-mutant AML exhibit an immunosuppressive microenvironment which can be exploited with immunotherapy. Studies showed that increased PD-L1 expression is an independent negative prognostic marker and that higher PD-L1 expression during treatment with HMA for AML correlated with early progression [82]. This led to multiple studies with PD-1 or PD-L1 checkpoint inhibitors in combination with HMA, some of which have progressed to phase II. Zeidan et al., in the first randomized phase 2 study in this setting, administered azacitidine 75 mg/m^2^ on days 1 to 7 with or without a PD-L1 inhibitor (durvalumab 1500 mg) on day 1 of every 28-day cycle in 129 older newly diagnosed AML patients and demonstrated good tolerability [83]. Numerically higher grade 3 side effects like febrile neutropenia (35.9% vs. 22.6%) and pneumonia (23.4% vs. 12.9%) were noted in the experimental arm. The overall survival or overall response rates were not different between the two groups and found increasing PD-L2 expression in the experimental arm during therapy, which could have been the mechanism of resistance to PD-L1 therapy. Another phase 2 study used the PD-1 inhibitor Nivolumab with azacitidine and reported higher ORR than historical controls from their institute and noted that higher CD3+ cells in pre-therapy marrow correlated with response [84]. Dual combination immunotherapy with Ipilimumab and nivolumab with HMA [85] are ongoing. However, given the limited efficacy of checkpoint inhibitors approved for solid tumors in AML, novel agents are needed. Sabatolimab, a TIM-3 inhibitor, is currently in clinical development. TIM-3 plays a role in regulating the innate immune system. TIM-3 is expressed on immune cells and leukemic myeloid cells but not on normal hematopoietic stem cells [86]. During an oral presentation at the ASH meeting in 2021, the authors reported that 91 patients, 50 with high-risk MDS and 41 with newly diagnosed AML, were treated with sabatolimab and had an ORR of 57% in the MDS cohort and 40% in the AML cohort. They also report good tolerability with thrombocytopenia, neutropenia, and anemia as the only grade 3 side effects. A phase II study of sabatolimab in combination with azacitidine and Venetoclax in AML is active but not recruiting at this time (STIMULUS-AML1), NCT04150029.

## 12. Cell Therapy

Immunotherapy with adoptive/engineered T-cells called Chimeric Antigen Receptor (CAR)-T cells has had major breakthroughs in the treatment of Acute lymphoblastic leukemia [87] and diffuse large B cell lymphoma [88]. T cells are harvested from patients and engineered ex vivo to incorporate specific cell surface ligand molecules, which can then identify specific targets with these cell surface markers and redirect the specificity of T cells and lead to target cell killing. Engineered CAR-T cells have undergone many changes over the last few years and newer generation CAR-T cells are equipped with multiple costimulatory domains and can activate downstream transcription factors to induce cytokine production [89]. However, the lack of identification of a universal cell surface marker present only in blast cells that can be targeted without generating immunogenicity towards normal stem cells has meant that the AML story has not had a fairy tale ending yet. Many targets like CD123 [90], CD33, and C-type lectin-like molecule-1(CLL1) [91,92], CD38 [93], and approaches using a bispecific target [94]—CD13 and TIM3—are in the early stages of development. A common issue with these agents has been intense myelosuppression. Another unique challenge in AML has been the immunosuppressive tumor microenvironment that causes decreased T cell proliferation [95] or leads to early T cell exhaustion or decreased persistence [96]. Perna et al. have integrated proteomics and transcriptomic data to identify novel surface markers in AML, which can be used for engineering newer generations of CAR-T cells, and we eagerly look forward to this in the near future [97]. CAR-T cells with ON and OFF switches have already been tested in other diseases [98] and are awaited in AML and could lead to better toxicity profiles with lower myelosuppression. Natural Killer (NK) cells are another cell therapy modality that has been explored in AML. Autologous NK cells derived from the patient’s own body, HSCT donor-derived NK cells [99], adaptive NK cells [100] that expand in humans in response to human cytomegalovirus, and cytokine-induced memory-like NK [101] cells have all been tested in AML without significant efficacy. The ability to become activated without depending on antigen-presenting cells and the possibility of an off-the-shelf product without significant waiting times are attractive advantages that drive researchers to continue exploring different sources of NK cells as well as strategies to enhance their ability to identify specific targets [3]. Bispecific engagers of NK cells called BiKE [102] and trispecific killer cell engagers TriKE [103] are in the early stages of development and offer new hope.

## 13. Vitamin C

An essential vitamin for human survival, vitamin C has numerous functions in our body. It acts as a cofactor for numerous enzymes, some of which are involved in epigenetic modulation like ten eleven translocase-2 (TET2) [104]. Hematopoietic stem cells and brain cells contain some of the highest levels of vitamin C among human cells [105]. TET2 loss is one of the most common mutations encountered in healthy individuals as they age, now recognized as a precancerous condition called clonal hematopoiesis of indeterminate potential (CHIP), when no hematological manifestations are noted with the presence of these leukemia-related mutations [106]. Due to the many hematological symptoms that occur with vitamin C deficiency, historically scientists have explored the use of vitamin C in the treatment of leukemia even before their role in HSCs was found. Studies have looked at the synergy between vitamin C and a variety of FDA-approved agents including hypomethylating agents [107]. Recently our group defined the prevalence of vitamin C deficiency in myeloid malignancies and reported a trend toward a younger age of incidence of leukemia in this subpopulation [108]. Multiple clinical trials [NCT03682029] evaluating the use of vitamin C in preleukemic conditions like Clonal Cytopenia of Uncertain Significance (CCUS) to prevent these from progressing to leukemia are underway [109]. Vitamin C supplementation can retard leukemogenesis in mouse models [105] and researchers will need to define if there is a preleukemic subpopulation who might benefit from supplementation.

## 14. Conclusions

Many giant leaps in drug discovery and development for cancer over the past decade were made, and this has also had a positive influence on AML, with over 10 molecules now FDA-approved for this cancer over this period. Many more promising newer agents are currently in the nascent stages of development and provide new a ray of hope to our patients and newer tools at our disposal. Small molecule inhibitors, antibody-drug conjugates with targeted cytotoxicity, immunotherapy, cell therapy, and advances in stem cell transplants are leading to a paradigm shift in the way we risk stratify and treat acute myeloid leukemia. Survival among older patients has significantly improved with the addition of venetoclax to the backbone of HMA therapy, however, myelosuppression and infectious complications have to be monitored closely.

The recognition of preleukemic disease entities, Clonal Hematopoiesis of Indeterminate Potential (CHIP), and Clonal Cytopenia of Uncertain Significance (CCUS), and their inclusion in the WHO classification [110], will lead to more research interest in these entities and possibly lead to research on preventing leukemic transformations. We speculate that research groups will focus on interventional studies at this preleukemic stage to prevent transformation to leukemia and better define the groups with the highest risk of leukemic transformation and the various factors, including gut microbiota, which influence this progression [111,112].

There have been several unique challenges faced by researchers who work on this disease. AML is a disease of the older population, and we still do not have a clear system or prognostic tool to help identify individuals who are unable to tolerate traditional chemotherapy and should be considered upfront for other treatment modalities. The unique tumor microenvironment and heterogeneous nature of this disease have posed challenges to the development of chemo-free options including immunotherapy, targeted therapy, and cell therapy. Participation in clinical trials has been shown to improve outcomes, however, the recent pandemic had posed unique challenges to clinical trial accruement and participation [113]. Survival among older patients and poor-risk subgroups continues to be dismal in this disease and offering clinical trial options with promising new agents and combination strategies should always be considered. Novel agents in AML and drug development in the current state are reminiscent of this quote by Robert Frost “And miles to go before I sleep, and miles to go before I sleep.”

## Figures and Tables

**Table 1:** Selected phase 3 trials in AML.

NCT Number	Study Name	Study Design and Status	Drugs Tested	Drug Class	Primary Outcome
NCT02013648	Randomized Phase III Study of Intensive Chemotherapy with or Without Dasatinib	Randomized, open label Active not recruiting	Dasatinib	Tyrosine Kinase inhibitor	EFS
NCT04102020	Randomized, Double-Blind, 2-Arm, Multicenter, Phase 3 Study of Venetoclax and Oral Azacitidine Versus Oral Azacitidine as Maintenance Therapy for Patients with Acute Myeloid Leukemia in First Remission After Conventional Chemotherapy (VIALE-M)	Randomized, double blind Active and recruiting	Venetoclax Azacitidine CC-486	BCL2 inhibitor	1. DLT 2. DLT of combination 3. RFS
NCT04716114	A Phase 3, Open-Label, Multicenter, Randomized Study of SKLB1028 Versus Salvage Chemotherapy in Patients with FLT3-mutated Acute Myeloid Leukemia Refractory to or Relapsed After First-line Treatment (ALIVE)	Randomized, open label Active and recruiting	SKLB1028	Multikinase inhibitor of EGFR, FLT3 and ABL	1. CR/CRh rate 2. OS
NCT05586074	HEC73543 Versus Salvage Chemotherapy in Relapsed or Refractory FLT3-ITD Acute Myeloid Leukemia: a Multicenter, Open-label, Randomized Phase 3 Trial	Randomized, Open label Active not yet recruiting	Clifutinib	FLT3 inhibitor	OS CR/CRh rate
NCT04161885	A Randomized, Open Label Phase 3 Study Evaluating Safety and Efficacy of Venetoclax in Combination with Azacitidine After Allogeneic Stem Cell Transplantation in Subjects With Acute Myeloid Leukemia (AML) (VIALE-T)	Randomized, Open label Active, recruiting	Venetoclax Azacitidine	BCL2 inhibitor	DLT of combination RFS
NCT04628026	A Randomized, Placebo-Controlled Phase III Study of Induction and Consolidation Chemotherapy with Venetoclax in Adult Patients with Newly Diagnosed Acute Myeloid Leukemia or Myelodysplastic Syndrome with Excess Blasts-2	Randomized, double blind Active, recruiting	Venetoclax	BCL2 inhibitor	EFS DLT
NCT04571645	A Randomized, Double-blind, Placebo-controlled Study to Evaluate the Efficacy and Safety of Dociparstat Sodium in Combination with Standard Chemotherapy for the Treatment of Newly Diagnosed Acute Myeloid Leukemia	Randomized, double blind Active, not recruiting	Dociparastat	CXCR4/CXCL12 inhibitor	OS
NCT02997202	A Multi-center, Randomized, Double-blind, Placebo-controlled Phase III Trial of the FLT3 Inhibitor Gilteritinib Administered as Maintenance Therapy Following Allogeneic Transplant for Patients with FLT3/ITD AML	Randomized, double blind Active, not recruiting	Gilteritinib	FLT3 inhibitor	RFS
NCT05429632	Randomized, Double-blind, Placebo-controlled, Multi-center Phase III Study to Evaluate the Efficacy and Safety of Mocravimod as Adjunctive and Maintenance Treatment in Adult AML Patients Undergoing Allogeneic HCT	Randomized, double blind Active, recruiting	Macrovimod	Sphingosine-1-phosphate receptor modulator	RFS
NCT03258931	Phase III Randomized Study of Crenolanib Versus Midostaurin Administered Following Induction Chemotherapy and Consolidation Therapy in Newly Diagnosed Subjects With FLT3 Mutated Acute Myeloid Leukemia	Randomized, open label Active, recruiting	Crenolanib	FLT3 inhibitor	EFS
NCT04229979	A Randomized, Open-Label Study of the Efficacy and Safety of Galinpepimut-S (GPS) Maintenance Monotherapy Compared to Investigator’s Choice of Best Available Therapy in Subjects with Acute Myeloid Leukemia Who Have Achieved Complete Remission After Second-Line Salvage Therapy	Randomized, Open label Active, recruiting	Galinpepimut-S	WT-1 peptide vaccine	OS
NCT05079230	A Phase 3, Randomized, Double-Blind, Placebo-Controlled Study Evaluating the Safety and Efficacy of Magrolimab Versus Placebo in Combination with Venetoclax and Azacitidine in Newly Diagnosed, Previously Untreated Patients with Acute Myeloid Leukemia Who Are Ineligible for Intensive Chemotherapy	Randomized, Double blind Active, recruiting	Magrolimab	Anti-CD47	CR OS
NCT03616470	A Phase III Randomized, Double-Blind Trial to Evaluate the Efficacy of Uproleselan Administered with Chemotherapy Versus Chemotherapy Alone in Patients With Relapsed/Refractory Acute Myeloid Leukemia	Randomized, Double blind Active, not recruiting	Uproleselan	E-selectin antagonist	OS
NCT02668653	A Phase 3, Double-Blind, Placebo-controlled Study of Quizartinib Administered in Combination with Induction and Consolidation Chemotherapy, and Administered as Continuation Therapy in Subjects 18 to 75 Years Old with Newly Diagnosed FLT3-ITD (+) Acute Myeloid Leukemia (QuANTUM First)	Randomized, Double blind Active, not recruiting	Quizartinib	FLT3 inhibitor	OS
NCT03250338	Phase III Randomized, Double-blind, Placebo-controlled Study Investigating the Efficacy of the Addition of Crenolanib to Salvage Chemotherapy Versus Salvage Chemotherapy Alone in Subjects ≤ 75 Years of Age with Relapsed/Refractory FLT3 Mutated Acute Myeloid Leukemia	Randomized, double blind Active, recruiting	Crenolanib	FLT3 inhibitor	EFS
NCT04161885	A Randomized, Open Label Phase 3 Study Evaluating Safety and Efficacy of Venetoclax in Combination with Azacitidine After Allogeneic Stem Cell Transplantation in Subjects With Acute Myeloid Leukemia (AML) (VIALE-T)	Randomized, open label Active, recruiting	Venetoclax Azacitidine	BCL2 inhibitor	DLT RFS
NCT05020665	A Phase 3, Randomized, Double-blind, Placebo-controlled Study to Assess the Efficacy and Safety of Entospletinib in Combination With Intensive Induction and Consolidation Chemotherapy in Adults With Newly Diagnosed Nucleophosmin 1-mutated Acute Myeloid Leukemia	Randomized, double blind Active, not recruiting	Entospletinib	spleen tyrosine kinase (SYK) inhibitor	MRD negative CR
NCT04778397	A Phase 3, Randomized, Open-Label Study Evaluating the Safety and Efficacy of Magrolimab in Combination With Azacitidine Versus Physician’s Choice of Venetoclax in Combination With Azacitidine or Intensive Chemotherapy in Previously Untreated Patients With TP53 Mutant Acute Myeloid Leukemia ENHANCE-2	Randomized, Open label Active, recruiting	Magrolimab	Anti-CD47	OS
NCT02665065	A Multicenter, Pivotal Phase 3 Study of Iomab-B Prior to Allogeneic Hematopoietic Cell Transplant Versus Conventional Care in Older Subjects with Active, Relapsed or Refractory Acute Myeloid Leukemia (AML) (SIERRA)	Randomized, Open label Active, not recruiting	Iomab-B	Anti-CD45-^131^I apamistamab	dCR
NCT03926624	Phase 3 Randomized Trial of DFP-10917 vs. Non-Intensive Reinduction (LoDAC, Azacitidine, Decitabine, Venetoclax Combination Regimens) or Intensive Reinduction (High & Intermediate Dose Cytarabine Regimens) for Acute Myelogenous Leukemia Patients in Second, Third, or Fourth Salvage	Randomized, Open label Active, recruiting	DFP-10917	deoxycytidine nucleoside analogue	CR Duration of CR
NCT03268954	A Phase 3, Randomized, Controlled, Open-label, Clinical Study of Pevonedistat Plus Azacitidine Versus Single-Agent Azacitidine as First-Line Treatment for Patients with Higher-Risk Myelodysplastic Syndromes, Chronic Myelomonocytic Leukemia, or Low-Blast Acute Myelogenous Leukemia	Randomized, Open label Active, not recruiting	Pevonedistat	NAE inhibitor	EFS
NCT03092674	A Randomized Phase II/III Trial of “Novel Therapeutics” Versus Azacitidine in Newly Diagnosed Patients With Acute Myeloid Leukemia (AML) or High-Risk Myelodysplastic Syndrome (MDS), Age 60 or Older LEAP: Less-Intense AML Platform Trial	Randomized, Open label Active, not recruiting	Nivolumab	PD-1 inhibitor	OS
NCT03257241	A PALG Prospective Multicenter Clinical Trial to Compare the Efficacy of Two Standard Induction Therapies (DA-90 vs. DAC) and Two Standard Salvage Regimens (FLAG-IDA vs. CLAG-M) in AML Patients ≤ 60 Years Old	Randomized, Open label Active, recruiting	DNR 90 DAC CLAG-M FLAG- IDA	Anthracycline	Induction regimen efficacy Salvage regimen efficacy

EFS—Event free survival, DLT—Dose limiting toxicity, RFS- Relapse free survival, CR Complete Remission, CRh—Complete remission hematological, OS—Overall Survival, MRD—minimal residual disease, dCR—Durable Complete Remission.
